# Secondary oxidized di-2-ethylhexyl phthalate metabolites may be associated with progression from isolated premature thelarche to central precocious or early puberty

**DOI:** 10.1038/s41598-023-32768-1

**Published:** 2023-04-05

**Authors:** Xiuxin Zheng, Huiping Su, Shurong Huang, Wei Su, Rongfei Zheng, Yue Shang, Qiru Su, Li Zhou, Yao Yao, Zhe Su

**Affiliations:** 1 Department of Endocrinology, Shenzhen Children’s Hospital, Shenzhen, Guangdong China; 2 Department of Endocrinology, Fujian Children’s Hospital, Fuzhou, Fujian China; 3 School of Medicine, Southern University of Science and Technology, Shenzhen, Guangdong China; 4 Department of Clinical Research, Shenzhen Children’s Hospital, Shenzhen, Guangdong China; 5 Shenzhen Center for Disease Control and Prevention, Shenzhen, Guangdong China; 6 Central Laboratory, Longgang District Maternal and Child Healthcare Hospital, Shenzhen, Guangdong China

**Keywords:** Environmental social sciences, Endocrinology, Health care

## Abstract

Phthalate esters (PAEs) may act as estrogen receptor agonists, and their relationship with precocious puberty is a global health concern. However, their role in isolated premature thelarche (IPT) progression remains unclear. We conducted a cohort study investigating the relationship between IPT progression and urinary PAE metabolites. Girls with IPT aged 6–8 years were regularly followed up every three months for one year. Clinical data and urine PAE metabolite levels were collected. Participants who progressed to central precocious puberty (CPP) or early puberty (EP) had significantly higher ovarian volume, breast Tanner stage, and levels of the creatinine-adjusted urinary secondary oxidized di-2-ethylhexyl phthalate (DEHP) metabolites (Σ_4_DEHP). Breast Tanner stage (odds ratio [OR] = 7.041, *p* = 0.010), ovarian volume (OR = 3.603, *p* = 0.019), and Σ_4_DEHP (OR = 1.020, *p* = 0.005) were independent risk factors for IPT progression. For each 10 µg/g/Cr increase in the urine level of Σ_4_DEHP, the risk of progression from IPT to CPP/EP within one year increased by 20%. This study demonstrated that the breast Tanner stage, ovarian volume, and Σ_4_DEHP in urine were independent risk factors for IPT progression, and Σ_4_DEHP may be associated with the progression of IPT to CPP or EP.

## Introduction

*Isolated premature thelarche (IPT)* is defined as unilateral or bilateral breast development in girls before age eight^1^. IPT appearing before the age of 2–4 may be attributable to mini-puberty^2^. However, IPT presented at an older age (mostly after age six) may progress to central precocious puberty (CPP) or early puberty (EP)^3^. These conditions may have a detrimental effect on the final adult stature and psychology^4^. At present, relevant studies have mainly focused on indicators of clinical transformation, such as serum levels of estradiol, luteinizing hormone (LH)^5^, insulin-like growth factor-1 (IGF-1) standard deviation score (SDS)^6^, age at onset^7^, absolute growth velocity^5^, weight^8^ and estrogen effects on target organs, such as uterus length and breast Tanner stage^3^. However, the results varied, and few studies have explored the underlying mechanism.

Exposure to phthalate esters (PAEs) is of great concern^9^, and PAEs are omnipresent: in food, water, breast milk, soil, breathing air, dust, dress materials, dwellings, hospitals, jails, etc.^10^. Di-2-ethylhexyl phthalate (DEHP), diethyl phthalate (DEP), di-n-butyl phthalate (DBP), and di-isobutyl phthalate (DiBP) are the four PAEs with relatively high detection levels in indoor environments, river sediments, and aquaculture fishponds in the Pearl River Delta region, where Shenzhen is located^11–13^. Furthermore, the levels of PAEs in water and sediments in the Pearl River Delta Basin recently showed an increasing trend^13–15^. More importantly, the metabolites of these four PAEs are also higher than those of other PAE types in the urine of school students in Shenzhen, with a detection rate of 80–100%^16^, indicating that children in Shenzhen have relatively high exposure to PAEs.

The relationship between PAEs and precocious puberty is a widespread concern. Different studies have shown significant associations with precocious puberty^17–19^, while no relationship with puberty^20^ or an association with the later onset of puberty^21,22^ has also been reported. A meta-analysis showed that DEHP and DEP may lead to precocious puberty^23^. Our published study presented that low-molecular-weight PAEs metabolites were associated with CPP by comparing the age-matched and BMI-matched groups of IPT, CPP, and girls with no breast development^24^. Although the mechanism remains to be further elucidated, endocrine-disrupting chemicals (including PAEs) might act through a number of mechanisms at some level in the hypothalamic-pituitary–gonadal (HPG) axis and peripheral endocrine organs^25^. For example, PAEs might act as estrogen receptor agonists or androgen receptor antagonists and might also interfere with androgen synthesis^26,27^. Therefore, we speculate that PAEs may be associated with progressing from IPT to CPP or EP. However, the role of PAEs in the progression of IPT has not been previously reported. Therefore, we conducted this cohort study to investigate the relationship between urinary metabolites of the four relatively high-exposure PAEs (DEHP, DEP, DBP, and DiBP) and the progression of IPT to CPP or EP in girls within one year.

## Methods

### Study population

Girls aged 6–8 years with isolated breast development underwent their first evaluation in the outpatient clinic of Shenzhen Children's Hospital from December 2018 to June 2019. They were hospitalized for a GnRH stimulation test when they presented with no breast tissue regression for at least three months and/or rapid progression in the breast Tanner stage, significantly advanced bone age, and/or linear growth acceleration. IPT was based on the following diagnostic criteria: peak LH level lower than 5 IU/L and ratio between peak LH and peak FSH in the GnRH stimulation test lower than 0.6. Meanwhile, uterine length was less than 3.5–4 cm, and ovarian volume was less than 2 ml^28,29^. All of the IPT girls had no pubic hair and armpit hair growth. Initially, 84 girls diagnosed with IPT were included in the study.

The exclusion criteria were as follows: other endocrine diseases (including growth hormone deficiency, thyroid dysfunction, adrenocortical dysfunction, or hyperactivity), neurological disorders (such as tumors, trauma, or malformations), local mammary gland hyperplasia, history of hormone treatment, history of nutritional tonic (such as cubilose, velvet antler, and others) and traditional Chinese medicine intake, family history of CPP, premature birth, and small size for gestational age. Ultimately, 74 participants were enrolled in this study. Clinical data and urine specimens examined for PAEs were collected from all the children at baseline.

This cohort study is registered as ChiCTR1900020492 in the Chinese Clinical Trial Registry (http://www.chictr.org/cn/). The study was approved by the Ethics Committee of Shenzhen Children's Hospital (No. 2018031). All girls participated voluntarily, and written consent was obtained from their parents.

### Research visit

Seventy-four participants with IPT were regularly followed up every three months for one year. We-Chat groups were formed and managed by qualified pediatric endocrinologists to improve compliance with the research visits. Participants were reminded of their follow-up appointments every month, and monthly IPT follow-up clinic visits were conducted. Height, weight, and pubertal stage were assessed at each visit. If the breast was regressive, patient observation continued. If the thelarche stage was persistent or progressive, the patients were reviewed for basal LH, FSH, estradiol, pelvic ultrasound, and bone age analyses if necessary. According to the values, they could be evaluated for further GnRH stimulation tests.

The outcome of this study was whether IPT progressed to CPP/EP within the one-year ± two months follow-up period. Following the Society of Pediatrics, Chinese Medical Association (2015) guidelines^30^, participants who were converted before the age of eight were diagnosed with CPP, and those who were converted at an age older than eight and younger than nine were diagnosed with EP. Exclusion criteria during follow-up were as follows: (1) patients who missed the follow-up or failed to cooperate during the follow-up period, (2) patients who received any sex hormone treatment, and (3) patients suffering from endocrine diseases, as mentioned above.

**Figure 1:**
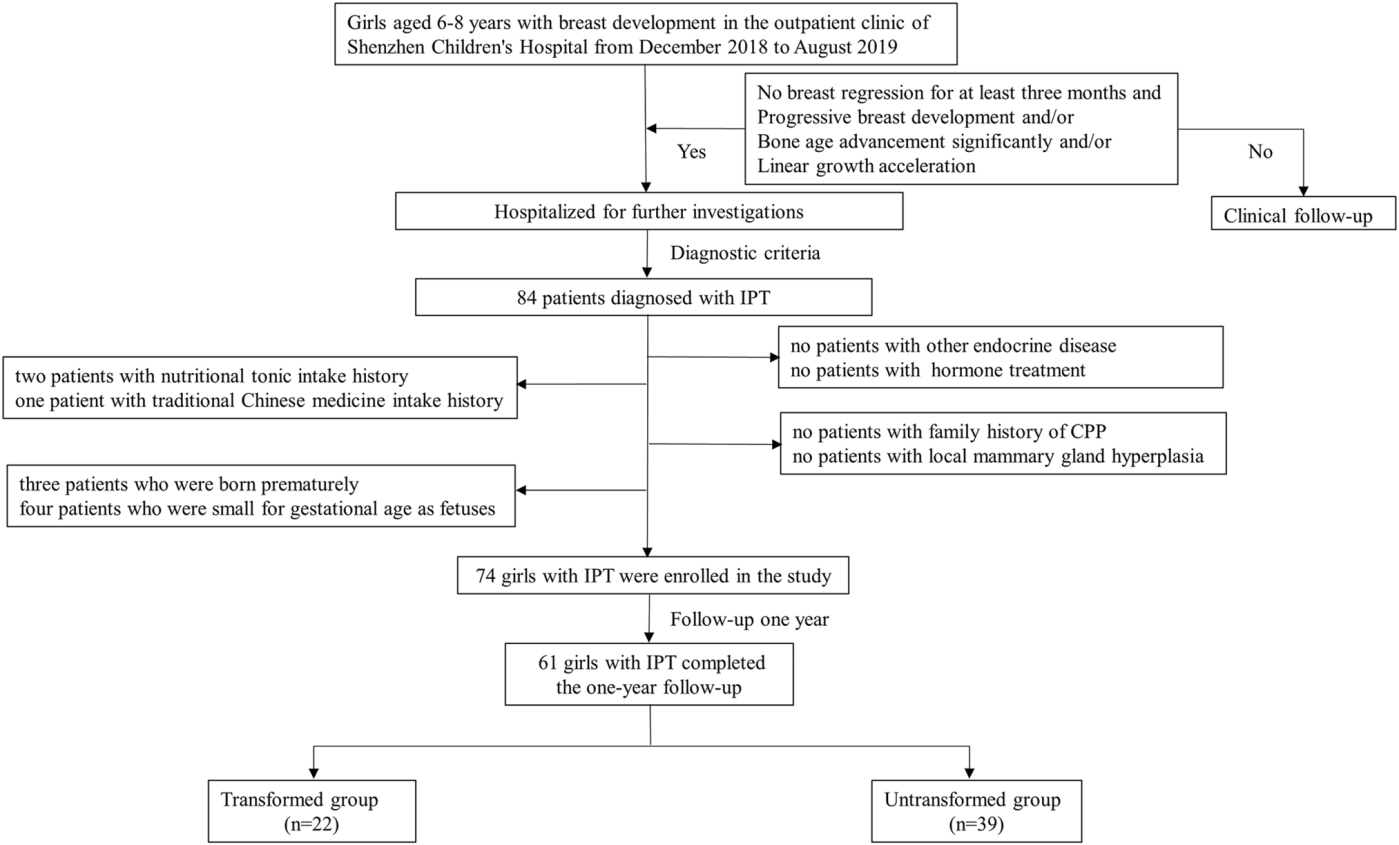
Flowchart of the study population selection process. *IPT* isolated premature thelarche, *CPP* central precocious puberty.

Patients were divided into two groups according to the follow-up results (Fig. 1). The transformed group comprised participants who progressed to CPP or EP. The untransformed group comprised participants with breast regression or no progression of breast development and clinical and biochemical characteristics (basal LH, FSH, estradiol, pelvic ultrasound, and bone age) during follow-up.

### Clinical data collection

Height and weight were measured, and the body mass index was calculated as body weight (kg)/height (m^2^). The values were expressed as age- and sex-specific SDS based on national survey data from 2005^31^. Two trained pediatric endocrinologists evaluated the staging of pubertal development according to the Tanner criteria^32^. A larger breast was the determinant of breast stage in the participants.

Levels of serum hormones, including LH, FSH, estradiol, dehydroepiandrosterone sulfate, and IGF-1, were measured at 8 am and 9 am using an electrochemiluminescence immunoassay (Siemens IMMULITE 2000, Munich, Germany). IGF-1 levels were standardized according to reference data for different age groups in China^33^. The lower limit of detection (LOD) for estradiol was 20 pmol/L, and data below 20 pmol/L were expressed as half of the lower LOD (10 pmol/L). The GnRH stimulation test (dose of 2.5–3 μg/kg, no more than 100 μg) was performed between 8 and 10 am. Plasma gonadotropin concentrations were determined at time zero and 30, 60, and 90 min after gonadorelin administration (Manshan Fengyuan, China).

Bone age was evaluated using the China 05 (CH05) method on a radiograph of the left hand^34^, the standard method for determining bone maturity in Chinese children based on the TW3 RUS method. Pelvic ultrasound evaluations were performed by an experienced pediatric radiologist using an ultrasound machine (GE logic E9) with a 3.5–12 MHz probe and a 9–12 MHz linear probe. Ovarian volume was calculated using the formula for a prolate ellipsoid and the mean of the right and left ovaries.

### Urinary phthalate metabolite sampling and analysis

The participants' first-morning urine sample was collected in a fasted state. Urine samples were collected in polypropylene tubes (50 mL) and transported to the laboratory at the Shenzhen Center of Disease Control and Prevention on ice within 4 h. Urine samples were stored at − 40 °C and tested within one year.

Eight PAE metabolites were assessed in this study: monoethyl phthalate (MEP), mono-n-butyl phthalate (MBP), monoisobutyl phthalate (MiBP), mono(2-ethyl-5-hydroxyhexyl) phthalate (MEHHP), mono-(2-ethyl-5-oxohexyl) phthalate (MEOHP), mono-[(2-carboxy methyl)hexyl] phthalate (MCMHP), mono(5-carboxy-2ethylpentyl) phthalate (MCEPP), and mono-2-ethylhexyl (MEHP). Among these, MEHP is the primary metabolite of DEHP. Secondary oxidized DEHP metabolites (Σ_4_DEHP) include MEHHP, MEOHP, MCMHP, and MCEPP. Σ_4_DEHP means the summation of the four secondary oxidized DEHP metabolites.

PAEs with side chain lengths of 1–4 carbons are classified as low molecular weight (LMW)-PAEs, and those with side chain lengths of more than four carbons are classified as high molecular weight (HMW)-PAEs. LMW is the sum of three LMW-PAE metabolites (MEP, MBP, and MiBP), and HMW is the sum of the concentrations of five DEHP metabolites. All urinary PAE metabolites were analyzed using ultra-performance liquid chromatography and tandem mass spectrometry (UHPLC-MS/MS), according to a previously reported method^16^. Enzymatic deconjugation, automatic solid phase extraction, and instrumental analysis were the major analytical procedures. An AB Sciex Q-Trap 5500 tandem mass spectrometer (Applied Biosystems, Foster City, CA, USA) coupled with a Shimadzu 20AD UHPLC system was used in instrumental analyses of PAE metabolites. Quality control samples were analyzed for each batch of 40 samples, including two program blanks and one matrix-spiked sample. We subtracted the mean concentration of the program blanks from the final concentration of PAE metabolites for each specific batch. The concentrations of the PAE metabolites below the LOD were replaced with 1/2 LOD. The LOD of PAE metabolites ranged from 0.1 to 0.3 μg/L. Urinary creatinine was analyzed using UHPLC-MS/MS to correct urine dilution.

### Sample size

Sample size calculations were performed using G*Power (version 3.1.9.7; Germany). According to previous clinical observational studies and literature reports, the proportion of p1 was 30% (the highest ratio of IPT progression that we had searched)^8^, and that of p2 was 13% (the lowest one of IPT progression)^35^. With 90% power and 5% significance, the total sample size was 61, and the actual power was 90.8%. Based on a 20% loss rate, 76 cases should be included. However, a total of 74 participants were included in the study. After various measures to improve compliance, 61 patients completed the follow-up, which met the sample size requirements.

### Statistical methods

Statistical analyses were performed using IBM SPSS version 26.0 (IBM Cor. A *p*-value of < 0.05 was considered statistically significant for all analyses. Numerical variables are presented as means ± standard deviations or medians and quartiles. Categorical variables are presented as numbers and percentages. The independent samples *t*-test assessed the differences in numerical variables between the two groups when parametric test assumptions were met. In contrast, the Mann–Whitney U test was used when they were unmet. The association between significant bone age advancement and the status of obesity or being overweight was calculated using the chi-square test.

To identify independent risk factors that significantly affected IPT progression, we performed a binary logistic regression analysis (forward likelihood ratio stepwise method) with progression from IPT to CPP/EP (yes/no) as the dependent variable. The covariates were those shown to be significant (*p* < 0.05) in the comparison between the two groups (ovarian volume, breast Tanner stage, and Σ_4_DEHP) and other factors that may be clinically correlated (basal LH, uterus length, and serum IGF-1 SDS).

### Ethics approval

This cohort study was registered as ChiCTR1900020492 in the Chinese Clinical Trial Registry (http://www.chictr.org/cn/) on January 2, 2019. The study was performed in line with the Declaration of Helsinki and approved by the Ethics Committee of Shenzhen Children's Hospital (No. 2018031).

### Informed consent statement

Informed consent was obtained from all individual participants included in the study.

## Results

### Participant characteristics at baseline

Among the 74 participants, 61 (82.4%) completed the one-year follow-up period. As shown in Table 1, there were no differences in the clinical and laboratory characteristics between the initial participants and those who completed the follow-up. Among the 61 patients, 41 (67.2%) had breast Tanner stage 2, while the rest had breast Tanner stage 3. The bone age was advanced by 2.20 (1.40, 2.58) years compared to the chronological age.

**Table 1:** Participant characteristics.

Participant characteristics	Initial participants (n = 74)	Follow-up population (n = 61)	*p*-value
Age of onset (year)	7.45 (7.02, 7.72)	7.42 (7.00, 7.75)	0.85
Chronological age (year)	8.02 (7.63, 8.32)	8.03 (7.58, 8.35)	0.97
BMI SDS	0.65 ± 1.07	0.78 ± 1.06	0.90
Breast tanner stage			0.74
2	51 (68.9%)	41 (67.2%)	
3	23 (31.1%)	20 (32.8%)	
Basal LH (mIU/mL)	0.28 (0.15, 0.46)	0.27 (0.14, 0.46)	0.84
Basal FSH (mIU/mL)	3.19 (2.04, 4.59)	3.18 (2.18, 4.43)	0.82
Peak LH (mIU/mL)	5.43 (3.71, 7.41)	5.24 (3.70, 7.23)	0.89
Peak FSH (mIU/mL)	16.03 (13.07, 19.25)	16.30 (12.99, 19.28)	0.98
Estradiol (pmol/L)	10.00 (10.00, 23.25)	10.00 (10.00, 22.50)	0.70
IGF-1 SDS	0.20 (−0.46, 0.80)	0.16 (−0.42, 0.86)	0.76
BA-CA (year)	2.01 (1.40, 2.57)	2.20 (1.40, 2.58)	0.77
Uterus length (cm)	2.10 (1.80, 2.30)	2.10 (1.08, 2.30)	0.76
Ovarian volume (cm^3^)	1.42 (1.00, 1.87)	1.46 (0.99, 1.91)	0.70

*BMI* Body mass index, *SDS* Standard deviation score, *LH* Luteinizing hormone, *FSH* Follicle-stimulating hormone, *IGF-1* Insulin-like growth factor-1, *BA-CA* Bone age minus chronological age.

### Participant characteristics in the transformed and untransformed groups

There were 22 (36.1%) participants in the transformed group and 39 (63.9%) in the untransformed group. Compared with the untransformed group, the transformed group had a higher ovarian volume (*p* = 0.032) and breast Tanner stage (*p* = 0.043). There were no differences in chronological age; the levels of basal LH, follicle-stimulating hormone (FSH), and IGF-1 SDS; or uterine length between the two groups (Table 2).

**Table 2:** Participant characteristics in the transformed and untransformed groups.

	Transformed group (n = 22, 36.1%)	Untransformed group (n = 39, 63.9%)	*p*-value
Age of onset (year)	7.32 (6.75, 7.76)	7.47 (7.12, 7.76)	0.338
Chronological age (year)	8.17 (7.52, 8.39)	7.95 (7.59, 8.29)	0.336
Chronological age ≥ 8 year	8.27 (8.04, 8.43)	8.21 (8.11, 8.40)	0.622
Chronological age < 8 year	7.38 (7.16, 7.75)	7.63 (6.93, 7.77)	0.859
BMI SDS	0.78 ± 0.81	0.77 ± 1.19	0.946
BMI SDS ≥ 1	8 (36.4%)	17 (44.6%)	0.580
Breast tanner stage			0.043
2	11 (50.0%)	30 (76.9%)	
3	11 (50.0%)	9 (23.1%)	
Basal LH (mIU/mL)	0.35 (0.15, 0.88)	0.26 (0.11, 0.43)	0.247
Basal FSH (mIU/mL)	3.53 (2.49, 5.03)	3.04 (2.12, 4.30)	0.480
Peak LH (mIU/mL)	5.94 (4.26, 7.23)	4.76 (3.08, 7.27)	0.167
Peak FSH (mIU/mL)	13.99 (11.67, 18.12)	17.18 (13.54, 19.80)	0.050
Estradiol (pmol/L)	10.00 (10.00, 26.00)	10.00 (10.00, 22.00)	0.562
DHEAS (μg/dl)	45.80 (27.05, 66.25)	46.75 (26.50, 78.33)	0.800
IGF-1 SDS	0.13 (−0.31, 0.81)	0.23 (0.19, 1.18)	0.804
BA-CA (year)	2.10 (1.38, 2.60)	2.20 (1.40, 2.56)	0.735
BA-CA > 1 (year)	20 (90.9%)	35 (89.7%)	0.880
Uterus length (cm)	2.10 (1.90, 2.30)	2.00 (1.80, 2.30)	0.738
Ovarian volume (cm^3^)	1.41 (0.99, 1.70)	1.31 (0.86, 1.62)	0.032

*BMI* Body mass index, *SDS* Standard deviation score, *LH* Luteinizing hormone, *FSH* Follicle-stimulating hormone, *DHEAS* Dehydroepiandrosterone sulfate, *IGF-1* Insulin-like growth factor-1, *BA-CA* bone age minus chronological age.

### Urinary phthalate metabolite analysis

The detection rates of PAE metabolites in the urine of the 61 participants were as follows: MBP (100%), MECPP (100%), MiBP (100%), MEHHP (100%), MEP (93.4%), MEHP (91.8%), MEOHP (88.5%), and MCMHP (86.8%). The creatinine-adjusted urinary MCEPP, MEHHP, MEOHP, and MCMHP levels were significantly higher in the transformed group than those in the untransformed group (*p* < 0.05). However, the other creatinine-adjusted urinary PAE levels were not significantly different between the two groups. The details are listed in Table 3.

**Table 3:** Differences in PAE levels between the transformed and untransformed groups.

PAEs		Transformed group (n = 22, 36.1%)	Untransformed group (n = 39, 63.9%)	*p*-value
LMW (μg/g/Cr)		180.44 (106.85, 306.7)	201.21 (103.77, 568.29)	0.631
	MEP	11.77 (2.83, 26.15)	8.80 (4.42, 29.03)	0.869
	MiBP	16.49 (11.26, 39.23)	29.47 (1.41, 70.38)	0.470
	MBP	152.91 (59.49, 224.08)	145.64 (69.0, 342.39)	0.663
HMW-DEHP (μg/g/Cr)		48.06 (27.77, 104.09)	35.92 (19.31, 56.60)	0.099
Secondary	MEHHP	13.90 (7.29, 34.06)	6.81 (4.23, 15.01)	0.017
	MEOHP	8.04 (5.09, 17.11)	4.81 (2.56, 9.61)	0.017
	MCMHP	4.84 (3.12, 18.65)	3.01 (1.79, 5.94)	0.031
	MCEPP	15.04 (7.41, 28.87)	7.75 (4.26, 16.44)	0.037
Primary	MEHP	6.52 (2.67, 10.33)	4.60 (216, 10.00)	0.392

*PAEs* Phthalate esters, *LMW* Low molecular weight, *MEP* Monoethyl phthalate, *MiBP* Monoisobutyl phthalate, *MBP* Mono-n-butyl phthalate, *HMW-DEHP* High molecular weight di-(2-ethylhexyl) phthalate, *MEHHP* Mono (2-ethyl-5-hydroxyhexyl) phthalate, *MEOHP* Mono-(2-ethyl-5-oxohexyl) phthalate, *MCMHP* Mono-[(2-carboxy methyl) hexyl] phthalate, *MCEPP* Phthalatemono (5-carboxy-2ethylpentyl) phthalate, *MEHP* Mono-2-ethylhexyl.

### Independent risk factors affecting IPT progression

The results of binary logistic regression analysis (forward LR stepwise method) are presented in Table 4. Breast Tanner stage (odds ratio [OR] = 7.041, *p* = 0.010), ovarian volume (OR = 3.603, *p* = 0.019), and Σ_4_DEHP (OR = 1.020, *p* = 0.005) were independent risk factors for IPT progression. Compared to IPT with breast Tanner stage 2, IPT with breast Tanner stage 3 had a 7.041-fold higher risk of progressing to CPP/EP within one year. For each 1 mL increase in the ovarian volume and 10 µg/g/Cr increase in the urine level of Σ_4_DEHP, the risk of progression from IPT to CPP/EP within one year increased by 260% and 20%, respectively.

**Table 4:** Independent risk factors affecting progression of isolated premature thelarche.

	OR	95% CI	*p*-value
Breast tanner stage			
2	1.000		
3	7.041	1.595–31.094	0.010
Ovarian volume	3.603	1.236–10.504	0.019
Σ_4_DEHP	1.020	1.006–1.036	0.005
Uterus length	0.392	0.043–3.558	0.405
Basal LH	0.988	0.185–5.277	0.988
IGF-1 SDS	0.869	0.469–1.607	0.654

*Σ*_*4*_*DEHP* MEHHP [mono (2-ethyl-5-hydroxyhexyl) phthalate] + MEOHP [mono-(2-ethyl-5-oxohexyl) phthalate] + MCEPP [phthalatemono (5-carboxy-2ethylpentyl) phthalate] + MCMHP {mono-[(2-carboxy methyl) hexyl] phthalate}, *LH* Luteinizing hormone, *IGF-1* Insulin-like growth factor-1, *SDS* Standard deviation score, *OR* Odds ratio, *CI* Confidence interval.

## Discussion

This is the first cohort study investigating the relationship between IPT progression and urinary PAE metabolites. Urine PAEs were detected using UHPLC-MS/MS and corrected by creatinine to reduce the error caused by the urine concentration in the test. Our results showed that among the 61 participants with IPT, 22 (36.1%) progressed to CPP/EP during the one-year follow-up period. Breast Tanner stage, ovarian volume, and Σ_4_DEHP in urine were independent risk factors for the progression of IPT in the present cohort.

The reported rates of IPT progression to CPP vary. In a Chinese retrospective cohort study, Zhu et al.^3^ found that 21.5% of participants with IPT converted to CPP at a chronological age of 7.1 + / − 0.7 years. In our study, the ratio of IPT progression was 36.1%, which is also higher than that in the studies of Lee et al. (19.0%, in Taiwan, younger than six years)^36^, Volta et al. (18.4%, in Italy, 0–8 years)^7^, de Vries L et al. (13%, in Israel, birth)^35^, Uçar et al. (29.0%, in Turkey, younger than three years)^5^, and Dilek Ç et al. (29.7%, including rapidly progressive puberty, in Turkey, 0–8 years)^8^. These differences may be related to the inclusion criterion of age (onset at 6–8 years), which excluded any interference by mini-puberty and might have increased the probability of progression. In addition, the criteria requiring further investigation indicate that the breast might progress easily.

In contrast to previous studies^7,8,36,37^, this study presented the independent risk of breast Tanner stage and ovarian volume in IPT progression. Estrogen target organs are also associated with estrogen exposure. Long-term exposure to estrogen levels higher than prepubertal levels may be important in converting IPT to CPP. Similar to congenital adrenal hyperplasia and McCune–Albright syndrome, higher exposure to estrogen is highly susceptible to the transition from peripheral precocious puberty to CPP^3^. However, de Vries et al.^35^ showed that the levels of estradiol and adrenal hormones had no relationship with IPT progression to CPP, which was similar to the results of the present study. This may be associated with the volatility of estradiol itself, and different batches and testing methods could affect the test results, resulting in poor comparability. Thus, more attention should be paid to girls with IPT with higher breast Tanner stage and ovarian volume during the first visit and follow-up period.

In addition, the most important finding of the present study is that Σ_4_DEHP levels in the urine are independent risk factors for IPT progression. For each 10 µg/g/Cr increase in the Σ_4_DEHP level, the risk of progression from IPT to CPP/EP within one year increased by 20%. DEHP, an estrogenic environmental endocrine disruptor, is widely used as a plasticizer to manufacture polyvinyl chloride^38^. Because it is not covalently bound to plastic, it is easily emitted into the environment. The human body can absorb DEHP through the skin, gastrointestinal tract, respiratory tract, and other ways^39^. In prepubertal children, the clearance rate of estradiol metabolism is considered low. Therefore, exposure to estrogen-like chemicals may have significant biological effects on breast development prior to puberty.

DEHP may be associated with the progression of IPT to CPP through various central HPG axis and peripheral pathways. By gavage of DEHP in rats, DEHP was found to promote the release of gonadotropin-releasing hormone (GnRH) by interfering with the IGF-1/PI3K/Akt/mTOR signaling pathway in the hypothalamus, thereby affecting the function of the HPG axis and finally resulting in precocious puberty^40^. Moreover, DEHP may act as an obesogen and alter metabolic programming during fetal and early childhood development, resulting in alterations in the metabolic and peripheral hormones associated with the onset of puberty^27^. Intermittent fasting can ameliorate precocious puberty induced by DEHP exposure via the HPG axis^41^. As IPT is caused by the partial activation of the HPG axis, DEHP may promote the progression of IPT to CPP via the HPG axis. Studies have shown that PAEs can exert estrogen-like functions by binding to estrogen receptors^42^ and increasing estrogen synthesis by inducing aromatase gene expression^43^. Breasts and gonads are estrogen-target organs; therefore, DEHP may act directly on these organs to promote breast and ovarian development. More in-depth research is needed to reveal the possible mechanism of DEHP in the progression of IPT to CPP/EP.

DEHP is extensively metabolized after all routes of uptake. In the first and fastest step, DEHP is metabolized into primary MEHP and further metabolized by different oxidation reactions. Σ_4_DEHP exhibited considerably longer elimination half-lives (10–24 h vs. 4 h) and later maxima of urinary excretion than the simple monoester MEHP^44^. Thus, the Σ_4_DEHP levels in urine were several times higher than those of MEHP after DEHP intake for 24 h. These represent the major share of DEHP metabolites excreted in urine (70% for Σ_4_DEHP vs. 6% for MEHP)^44^. They are immune to external contamination and possibly the ultimate developmental toxicants^45^. Therefore, Σ_4_DEHP can reflect the exposure level of DEHP in the human body.

Some studies^46–48^ have shown that single urinary PAEs may reflect exposure levels for months or even years. Teitelbaum et al.^46^ collected 2–6 urine samples from each 6–10-year-old child within six months for environmental endocrine disruptor analysis, indicating that a single urine sample could moderately predict the average concentration of six months. Hauser et al.^47^ showed a moderate predictive effect on exposure to PAEs over three months in adult males. Similarly, a study^48^ revealed that DEHP levels in women's urine PAEs remained steady over 1–3 years (nurses). Also, the amount of PAEs in the environment that kids spend a lot of time in is rather steady. Examples include the furnishings and dust in homes and schools, water cups, skin care products, and other items that could be exposure sources^11^. If multiple urine samples from a single participant can be measured, the exposure level of PAEs may be more accurate^49^. Thus, our study has some limitations. First, the number of participants was small. Hence, expanding the sample size may yield more informative results. Second, collecting multiple urine samples from the same child during follow-up may have further increased the accuracy of PAE exposure.

## Conclusions

This study reveals that the breast Tanner stage, ovarian volume, and Σ_4_DEHP in urine are independent risk factors of IPT progression. In addition, Σ_4_DEHP may be associated with the progression of IPT to CPP or EP, which suggests that children with IPT should minimize exposure to DEHP.

## Data Availability

The datasets used and/or analysed during the current study available from the corresponding author on reasonable request.
